# Harnessing exceptional points for ultrahigh sensitive acoustic wave sensing

**DOI:** 10.1038/s41378-024-00864-5

**Published:** 2025-03-07

**Authors:** Xingyu Lu, Yang Yuan, Fa Chen, Xiaoxiao Hou, Yanlong Guo, Leonhard Reindl, Yongqing Fu, Wei Luo, Degang Zhao

**Affiliations:** 1 Department of Physics, Huazhong University of Science and Technology, Wuhan, China; 2 School of Integrated Circuits, Huazhong University of Science and Technology, Wuhan, China; 3 Institute for Microsystem Technology, Faculty for Engineering, University of Freiburg, Freiburg im Breisgau, Germany; 4 Faculty of Engineering and Environment, Northumbria University, Newcastle upon Tyne, NE1 8ST UK

**Keywords:** Physics, Electrical and electronic engineering, Materials science

## Abstract

Exceptional point (EP) is referred to degeneracies in a non-Hermitian system where two or more eigenvalues and their corresponding eigenvectors coalesce. Recently there have been significantly increased interests in harnessing EPs to enhance responsivities and achieve ultrasensitive detections in optics, electronics and acoustics, although there are few similar studies focused on using surface acoustic wave (SAW) sensing technologies, probably due to its great technical challenges. Herein, we proposed a scheme for accessing EPs in an on-chip architecture consisted of coupled-SAW-resonators system, forming a passive parity-time (PT) symmetric system. We demonstrated that by tuning additional losses in one of resonators and regulating the system in the proximity of the EP, the sensor exhibited significantly enhanced responses. As an example, we present an EP-based SAW gas sensor, which showed a much-improved sensitivity compared to that of a conventional delay-line SAW sensor. The fundamental mechanisms behind this excellent sensing performance have been elucidated.

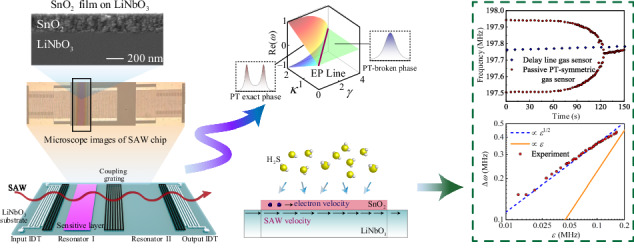

## Introduction

In a non-Hermitian physical system, exceptional points (EPs) are branch-point singularities in the parameter space where eigenvalues and their corresponding eigenvectors become coalesced. The parity-time (PT) symmetric systems, which is capable of exhibiting observable spectra, provide excellent platforms for realizing Eps^1–3^, Some significant developments for applying EPs have been made in realms of photonics^4–7^, electronics^8^, and acoustics^9–11^. EPs have received extensive attention for their great potentials to enhance the responsivity of sensors^12–22^. This topologically based enhanced sensing performance is based on the principle that in the proximity of an *N*th order (*N* = 2, 3, 4…) EPs, small perturbation $\varepsilon$ will lead to the significant detuning of frequencies, i.e., $\Delta \omega =|\omega -{\omega }_{EP}|\propto {\varepsilon }^{1/N}$. Even in the proximity of the second-order EP, the sensor often exhibits much higher sensitivity than the currently widely used linear frequency response sensor under small perturbations. Leveraging these characteristics of EPs has firstly been theoretically explored for enhancing sensing performance^23–25^, and then successfully been translated into some practical sensing applications^26^, most of which are based on either optical microcavities^15,27,28^ or electric circuits^12,13,29^.

For sensing applications, surface acoustic wave (SAW) devices have shown their great advantages such as compact size, digital output, compatibility with IC fabrication techniques, and ease of wireless passive sensing applications^30,31^. They are generally designed/fabricated based on MEMS technology, with advantages of integration and mass production. However, it has been a long-term challenge to maximize the sensing performance such as sensitivity or selectivity of the SAW based sensors. Therefore, it would be of great significance if applying these EPs in SAW platforms for achieving such the objective. However, there has few previous reports in literature for using these EPs for improving performance of SAW based sensing systems.

For the conventional SAW sensor, various sensitive films or layers have been deposited onto the piezoelectric substrate to improve the sensing ability^32^. Any alterations in mass, mechanical, or electric properties of these sensitive layers can affect the velocity of SAWs, consequently leading to shifts of center frequencies in transmission spectrum, which is the key mechanisms for SAW sensors. However, the sensitive layers also cause wave attenuation, primarily due to the acoustoelectric effect^33^. This naturally occurring dissipation spontaneously forms a divergent non-Hermitian system, and generates potential EPs, which can be exploited to enhance the responsivity of the SAW sensor.

In this study, to implement a PT-symmetric system to access EPs in an acoustic wave system, we use a coupled-SAW-resonators model with a sensitive thin film deposited on one of resonator to introduce additional losses, thus forming a passive PT-symmetric system^34–36^ In this case, we simply need to precise control the loss instead of introducing gain. By carefully adjusting the additional loss to render the system in the proximity of EPs, dramatic frequency changes in transmission peaks can be achieved to enhance the sensitivity and sensing performance. As an example of demonstration in this study, we investigated a SAW based hydrogen sulfide (${{\rm{H}}}_{2}{\rm{S}}$) gas sensor to confirm the feasibility of our proposed ideas. The developed sensor showed excellent sensing performance, such as rapid response to trace target gas, robustness to temperature variations, good selectivity and recoverability. Our proposed sensing mechanism offers a special sensing approach and proposes pioneering scheme applicable to SAW sensing systems, thereby opening up a new perspective for the development of the next-generation MEMS SAW sensing device.

## Results

### The passive PT-symmetric model on SAW chip

**Fig. 1:**
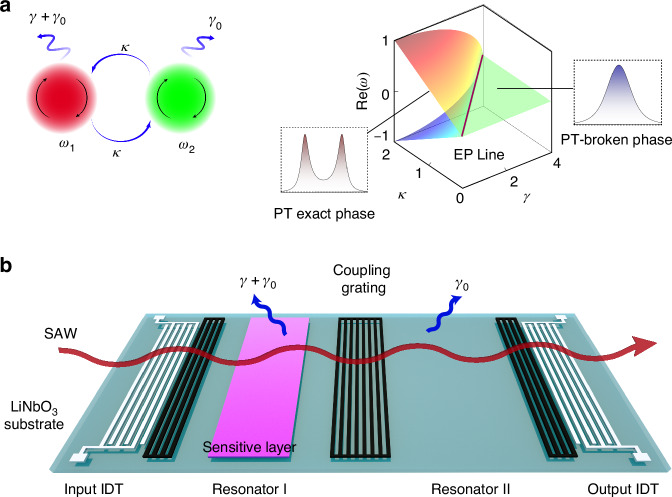
The passive PT-symmetric model for SAW sensor. **a** The schematic diagram of the coupled resonators model and the real part of the eigenfrequency surfaces in the parameter space (${\omega }_{1}={\omega }_{2}={\omega }_{0}=0$,${\gamma }_{0}=0$). The solid line denotes an EP line where $\gamma =2\kappa$. **b** A schematic diagram of the passive PT-symmetric SAW sensor. The coupled resonators are defined by three Bragg mirrors and coupled to the SAW transmission line. A sensitive layer is deposited onto the resonator I. External disturbance activates sensitive layer and introduce additional loss $\gamma$ in the resonator I

We first investigate a coupled-resonators model as shown in the left panel of Fig. 1a, in which ${\omega }_{1}$ and ${\omega }_{2}$ are the resonant frequencies of two resonators, and both of them possess symmetric inherent loss ${\gamma }_{0}$, while only one of them suffers an additional loss $\gamma$. The Hamiltonian of this system can be written as:

1 $${H}_{0}=\left(\begin{array}{cc}{\omega }_{1}-i{\gamma }_{0}-i\gamma & \kappa \\ \kappa & {\omega }_{2}-i{\gamma }_{0}\end{array}\right),$$

where $\kappa$ denotes the coupling strength between the two resonators. Under the condition ${\omega }_{1}={\omega }_{2}={\omega }_{0}$, the eigenfrequencies can be obtained as:

2 $${\omega }_{\pm }={\omega }_{0}-\frac{i}{2}(2{\gamma }_{0}+\gamma )\pm \frac{1}{2}\sqrt{4{\kappa }^{2}-{\gamma }^{2}}.$$

The evolution of the eigenfrequencies in the parameter space is shown in the right panel of Fig. 1a. For the case of $\gamma < 2\kappa$, the two eigenfrequencies have distinct real parts of ${\omega }_{0}\pm \sqrt{{\kappa }^{2}-{\gamma }^{2}/4}$, where the system is in the regime known as the “PT exact phase”. However, when $\gamma > 2\kappa$, the system enters the “PT-broken phase” and the real part of eigenfrequencies coincide to be a constant ${\omega }_{0}$^1^. At the critical state, $\gamma =2\kappa$, and the system exhibits an EP where two eigenvalues become coalesced. There is a corresponding relationship between the transmission peaks and the real parts of the eigenvalues of the system, specifically, the two transmission peaks merge into one single peak when the SAW system goes from PT exact phase to PT-broken phase. Such the dramatical variation in the observable spectra is the foundation of our proposed sensing mechanism. It should be also noted that the frequencies of the peaks are not equal to the eigenfrequencies^29,37,38^. More detailed analysis can be found in “Transmission Analysis” section.

Figure 1b shows the coupled resonators on a SAW chip which was made on a 128° Y-cut lithium niobite (${{\rm{LiNbO}}}_{3}$) substrate for strong electromechanical coupling and low propagation loss. The white gratings in Fig. 1b represent the input and output interdigital transducers (IDTs) with 60 aluminum finger electrodes. The coupled resonators are defined by three spaced reflecting gratings (or Bragg mirrors). The coupling strength $\kappa$ between these two resonators can be tuned by adjusting the number of electrodes in the middle Bragg mirror^39^. Therefore, to achieve a better coupling effect, the electrodes in the gratings and IDTs are made with identical widths and spacings. The pink plane in Fig. 1b represents a sensitive layer deposited onto one resonator. Upon exposure to external stimuli, additional loss $\gamma$ is introduced to drive the system towards accessing the EP.

### Transmission analysis

We define the intracavity mode fields of the resonators as ${a}_{1}$ (with the additional loss $\gamma$) and ${a}_{2}$ (without the additional loss), and the input field as ${a}_{in}$ and the output field as ${a}_{out}$. As the resonators are coupled to the SAW transmission line, it becomes necessary to introduce the coupling strength ${\gamma }_{c}$ to describe the interaction between the coupled resonator system and the transmission line. The dynamic equations for the coupled resonators SAW system are:

3 $$\begin{array}{l}\frac{d{a}_{1}}{dt}=-i{\omega }_{1}{a}_{1}-(\gamma +{\gamma }_{0}+{\gamma }_{c}){a}_{1}-i\kappa {a}_{2}-\sqrt{{\gamma }_{c}}{a}_{in}\\ \frac{d{a}_{2}}{dt}=-i{\omega }_{2}{a}_{2}-({\gamma }_{0}+{\gamma }_{c}){a}_{1}-i\kappa {a}_{1}\end{array}.$$

Substituting ${a}_{k}={A}_{k}{e}^{-i\omega t}$ and $\frac{d{a}_{k}}{dt}=-i\omega {A}_{k}{e}^{-i\omega t}+\frac{d{A}_{k}}{dt}{e}^{-i\omega t}$ and using input-output relation ${A}_{out}=\sqrt{{\gamma }_{c}}{A}_{2}$, the transmissivity of the system from input to output can be obtained as:

4 $$T(\omega )={|{S}_{21}|}^{2}={\left|\frac{\kappa {\gamma }_{c}}{{\kappa }^{2}+({\gamma }_{0}+{\gamma }_{c}+\gamma -i{\Delta }_{1})({\gamma }_{0}+{\gamma }_{c}-i{\Delta }_{2})}\right|}^{2}$$

where, ${\Delta }_{1}=\omega -{\omega }_{1}$ and ${\Delta }_{2}=\omega -{\omega }_{2}$ denote the frequency detuning. Setting $\Delta ={\Delta }_{1}={\Delta }_{2}$ (${\omega }_{1}={\omega }_{2}={\omega }_{0}$) and solving $dT/d\omega =0$, the frequencies of transmission peaks can be determined as:

5 $${\omega }_{\pm }=\left\{\begin{array}{c}{\omega }_{0}\pm \sqrt{{\kappa }^{2}-\frac{{\gamma }^{2}}{2}-\gamma ({\gamma }_{0}+{\gamma }_{c})-{({\gamma }_{0}+{\gamma }_{c})}^{2}},\,for\,\gamma \,\le \,\gamma^{\prime} \\ {\omega }_{0},\,for\,\gamma \,> \,\gamma^{\prime} \end{array}.\right.$$

**Fig. 2:**
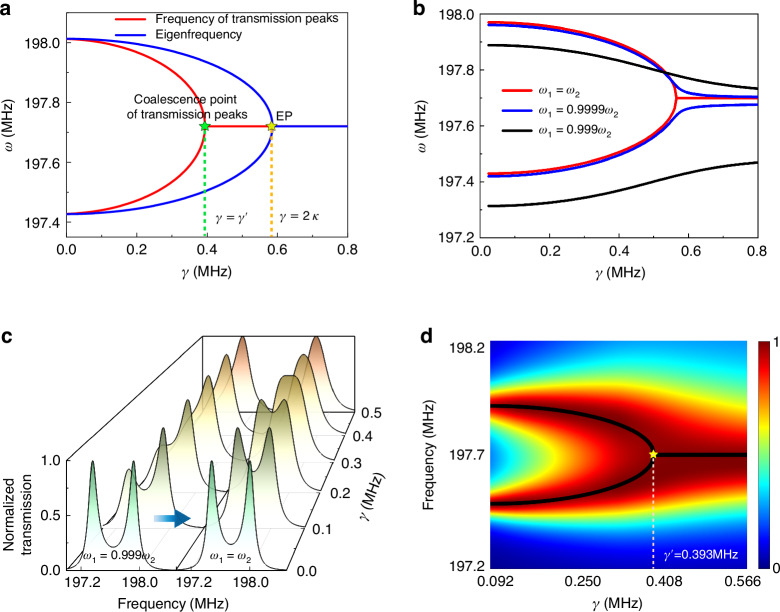
Evolution of SAW transmission. **a** The difference between the coalescence of transmission peaks and the coalescence of eigenfrequencies. **b** The evolution of the real part of the eigenfrequencies caused by the inconsistency of resonant frequencies. **c** The comparative evolution of transmission peaks of the coupled resonators system with $0.1 \%$ and without detuning of resonant frequencies of two resonators. **d** The transmission spectrum of the modified coupled resonators system with the increase of $\gamma$. The black solid curve roughly denotes the frequency position of transmission peaks. The yellow star is the coalescence point of transmission peaks

When $\gamma < \gamma^ {\prime}$, the transmission spectrum presents two peaks with their frequencies as ${\omega }_{+}$ and ${\omega }_{-}$, respectively. When $\gamma > \gamma ^{\prime}$, these two transmission peaks are merged into a single peak with a frequency of ${\omega }_{0}$. According to the Eq. (5), the critical state between two-peaks state and single-peak state is at $\gamma =\gamma ^{\prime}$, where $\gamma^ {\prime}$ is the solution of ${\kappa }^{2}-\frac{{{\gamma }^{\prime}}^ {2}}{2}-\gamma^ {\prime} ({\gamma }_{0}+{\gamma }_{c})-{({\gamma }_{0}+{\gamma }_{c})}^{2}=0$. It must be pointed out that this coalescence point of transmission peaks is obviously not the EP, i.e. $\gamma ^{\prime} =2\kappa$. Compared to eigenfrequency, the transmission peaks become coalesced before the EP, at the same frequency but with different $\gamma$, as shown in Fig. 2a. According to Eq. (4), we plot the Fig. 2a, with parameters assigned based on simulation results and the details can be found in the Experimental Section. This natural discrepancy makes the sensor not working at the EP, which can be applied to mitigate the noise owing to eigenbasis collapse at the EP^29,37,38^. Therefore, the appearance of such a single transmission peak does not imply the system is certainly being in the “PT-broken phase”.

It is important to note that the deposited sensitive thin film will slightly alter the resonant frequency of resonator due to its extra volume and mass loading effects. If ${\omega }_{1}\ne {\omega }_{2}$, the eigenfrequencies of Eq. (2) becomes:

6 $${\omega }_{\pm }=\frac{{\omega }_{1}+{\omega }_{2}-i\gamma -2i{\gamma }_{0}}{2}\pm \frac{1}{2}\sqrt{{({\omega }_{1}-{\omega }_{2})}^{2}+2({\omega }_{2}-{\omega }_{1})\gamma i+4{\kappa }^{2}-{\gamma }^{2}}.$$

Apparently, ${\omega }_{1}\ne {\omega }_{2}$ causes the annihilation of EPs and induce a baseline bifurcation of eigenfrequency^12^. EP is highly sensitive to detuning of ${\omega }_{1}$ and ${\omega }_{2}$, even when it is minute, for example, caused by the nanoscale thin film. As shown in Fig. 2b, only when 0.01% frequency detuning is introduced in ${\omega }_{1}$, with the increase of $\gamma$, the two eigenfrequencies are approached each other but not merged into degeneracy, i.e., no EP is existed. As the frequency detuning becomes more significant (e.g., reaching 0.1%), the passive PT-symmetric system is nearly transformed into a non-resonant coupled resonators system, which can be characterized by two distinct resonant frequencies^40^. Figure 2c shows the comparative results of the transmission peak’s evolution between the systems with and without EPs. Even when preserving subtle differences between ${\omega }_{1}$ and ${\omega }_{2}$, with the increase of additional loss $\gamma$, one peak experiences more significant decay and rapidly vanishes, whereas the other peak suffers much less loss and nearly maintain a constant magnitude. In addition, in the absence of EP, loss hardly influences the frequencies of the transmission peaks. All these details can be found in the Supplementary materials S1.

The EP-based SAW sensing is relied on the measurement of two peaks’ frequency difference^37^. That the disappearance of one peak (indicating that ${\omega }_{1}\ne {\omega }_{2}$) could lead to the invalidity of sensing functions. To address this issue, we added some electrodes in the rightmost Bragg mirror to compensate the frequency deviation caused by the deposition of the thin film. The number of added electrodes depends on the mechanical parameters of the sensitive layer. By combining the pre-experiment with thin film and simulated optimization, the merging behavior of the transmission peaks was achieved as expected, as shown in Fig. 2d. The coalescence point ($\gamma =\gamma^ {\prime} =0.393{\rm{MHz}}$) matches well with the theoretical result shown in Fig. 2a.

### Experimental implementation

**Fig. 3:**
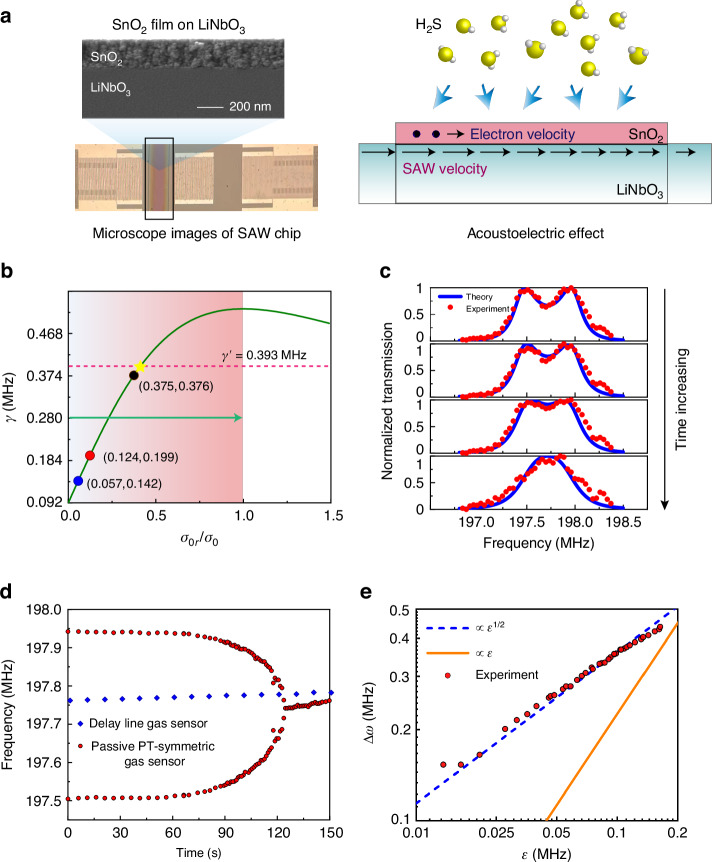
Experimentally measured responses of the sensor. **a** Photos of experimental samples and schematic diagram of additional loss caused by acoustoelectric effect. **b** The evolution process of the additional loss with the increase of sheet conductivity ratio ${\sigma }_{0r}/{\sigma }_{0}$. The marked points represent the initial states of three thin films (details can be found in Supplementary materials S3). **c** Transmission spectrum of our passive PT-symmetric SAW gas sensor as time increase. Red dots represent the experimental data and blue curves is the numerical fitting by employing the coupled mode theory. **d** The red dots represent the measured frequencies of the transmission peaks of passive PT-symmetric SAW gas sensor over time. The blue squares represent the measured center frequencies of the conventional delay line gas sensor over time. **e** The frequency splitting $\Delta \omega$ of passive PT-symmetric SAW gas sensor versus the perturbation $\varepsilon$ in a double-logarithmic scale. The blue dashed line and orange solid line indicates the square-root and linear scaling with perturbation $\varepsilon$, respectively

For our proposed method for harnessing EPs to achieve ultrasensitive sensing in the SAW systems, we chose ${{\rm{SnO}}}_{2}$ thin film as the sensitive layer and utilized the device to detect ${{\rm{H}}}_{2}{\rm{S}}$ gas as a demonstration. We have experimentally fabricated an on-chip SAW gas sensor sample to validate the feasibility of the aforementioned mechanisms, as shown in the left panel of Fig. 3a. The sensing effect is primarily based on the additional loss induced by the acoustoelectric effect of ${{\rm{SnO}}}_{2}$ thin film^33^. As shown in the right panel of Fig. 3a, when the target gas molecules are physically adsorbed onto the ${{\rm{SnO}}}_{2}$ thin film, the electron mobility of thin film is altered. When electrons travel in the direction of SAW propagation with a velocity less than that of the SAW, the SAW transfers kinetic energy to the electrons, resulting in the attenuation of the SAWs due to electron-acoustic wave interaction^41^. An equivalent RC circuit model was used to describe the attenuation of the SAW signals with respect to the wave intensity $I={I}_{0}{e}^{-\varGamma kx}$ along the propagation direction^42^:

7 $$\varGamma =\frac{{K}^{2}}{2}\frac{\frac{{\sigma }_{0r}}{{\sigma }_{0}}}{1+{(\frac{{\sigma }_{0r}}{{\sigma }_{0}})}^{2}},$$

where *K*^2^ = 5.5% is the electromechanical coupling coefficient for 128° Y-cut ${{\rm{LiNbO}}}_{3}$ substrate, ${\sigma }_{0}=1.67\times {10}^{-6}/\varOmega$ is the sheet conductivity of ${{\rm{LiNbO}}}_{3}$, ${\sigma }_{0r}$ is the sheet conductivity of ${{\rm{SnO}}}_{2}$ thin film, which is variable when the thin film absorbs the ${{\rm{H}}}_{2}{\rm{S}}$ gas molecules. Therefore, by injecting ${{\rm{H}}}_{2}{\rm{S}}$ gas onto the device, we can tune the attenuation of the SAWs in system through tuning the sheet conductivity of ${{\rm{SnO}}}_{2}$ according to Eq. (7). It should be noted that the SAW attenuation $\varGamma$ is not the additional loss $\gamma$ in Hamiltonian. The relation between them is $\gamma =31.5346\varGamma +0.09166\,({\rm{MHz}})$, which can be determined by numerical fitting of pre-experimental results of the individual thin film. The details can be found in the Supplementary materials S2.

As depicted in Fig. 3b, the values of $\gamma$ are increased with the increase of ${\sigma }_{0r}/{\sigma }_{0}$ from zero, reaching its maximum value of 0.525 MHz at ${\sigma }_{0r}/{\sigma }_{0}=1$, and are subsequently decreased afterwards. In the experiments, the necessary condition to observe two-peaks-to-one-peak process in transmission spectrum is set as that $\gamma$ is gradually increased from less than $\gamma ^{\prime}$ to greater than $\gamma^ {\prime}$. $\gamma^{\prime}$ should not be larger than the maximum $\gamma ^{\prime}$ value ($0.525{\rm{MHz}}$). In addition, $\gamma^ {\prime}$ should not be too small because it should be greater than the initial loss brought by the ${{\rm{SnO}}}_{2}$ thin film without injection of ${{\rm{H}}}_{2}{\rm{S}}$ gas. An appropriate value of $\gamma ^{\prime} =0.393{\rm{MHz}}$ was determined through simulation by carefully tuning the structural parameters. The ${{\rm{SnO}}}_{2}$ thin film was deposited using the magnetron sputtering technique, exhibiting an initial sheet conductivity which introduced an initial loss. This initial sheet conductivity of the thin film is predominantly dependent on the sputtering time. We conducted many pre-experiments to determine the appropriate sputtering time, with details available in Supplementary materials S3. If the initial sheet conductivity is too high, the variation range for the additional loss will be reduced. Conversely, if the initial sheet conductivity is too low, the reaction time for detecting tiny concentration of ${{\rm{H}}}_{2}{\rm{S}}$ gas becomes too long. To balance these issues, we selected the sputtering time of 45 min as the optimized condition, where the initial ${\sigma }_{0r}/{\sigma }_{0}$ is 0.124 and the initial additional loss is fitted as 0.199 MHz We then fabricated the SAW sensor sample according to the determined structural parameters and measured the transmission spectra by introducing $0.4\,\rm{ppm}\,{{\rm{H}}}_{2}{\rm{S}}$ gas. As is shown in Fig. 3c, if the additional loss is insignificant, two apparent separated peaks can be observed in the transmission spectrum. As the value of $\gamma$ is gradually increased, the split peaks are gradually merged into a single peak, which exhibits excellent agreement between the experiment and theory results.

The common sensing strategy of most conventional SAW gas sensors is measuring the center frequency shift of spectrum caused by disturbance from target gas. To highlight the advantages of our sensor, we also fabricated a SAW delay line gas sensor, by simply removing all the Bragg mirrors from our passive PT-symmetric SAW gas sensor. The details are schematically shown in Fig. S4a in the Supplementary materials S4. Both these two types of sensors were deposited with ${{\rm{SnO}}}_{2}$ thin films of the same width and thickness at the same location to introduce same disturbance in the environment with the same concentration of ${{\rm{H}}}_{2}{\rm{S}}$ gas. As shown in Fig. 3d, we recorded the frequency changes versus the increase of $\gamma$ of these two types of sensors for comparisons. Apparently, in the proximity of the coalescence point, the frequencies of transmission peaks of our developed passive PT-symmetric sensor have been dramatically changed in a very short time. In contrast, the frequency drift over time for the conventional delay line gas sensor was quite small, making the measurement inaccurate and challenging. In addition, it should be noted that the frequency at which the transmission peaks are merged is 197.742 MHz, which is a little different from the center frequency of initial double peaks (197.723 MHz). Besides, compared to Fig. 2a, after the two peaks are merged into a single peak, the frequency of the single peak is shifted slightly as time increases. All these small offsets are mainly caused by the changes in wave velocity due to the mass loading and elastic loading effects. The detailed analysis about these offsets can be found in the Supplementary materials S5. The transmission peak degeneracy has not been changed apparently by these offsets, which demonstrates the good performance of our sensor.

**Fig. 4:**
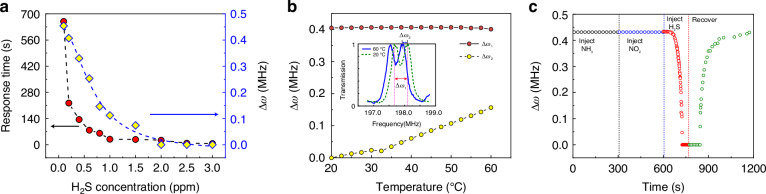
The sensing performance of sensor. **a** The sensing performance of the sensor with different concentrations of ${{\rm{H}}}_{2}{\rm{S}}$ gas. The red dots represent the average response times from two split transmission peaks changing into a single peak. The yellow quadrilaterals represent the frequency differences between two split transmission peaks at a fixed response time of 20 s. The blue dashed line is the fitting calibration curve of our sensor for ${{\rm{H}}}_{2}{\rm{S}}$. **b** The global frequency shift of two peaks (blue solid dots, $\Delta {\omega }_{2}$) and the frequency difference of two peaks (red solid dots, $\Delta {\omega }_{1}$) at different temperatures. Insert visually reveals the global frequency drift phenomenon at $20^{\circ} C$ and ${60}^{\circ} C$. **c** Response of sensor to the mixed gas with sequentially injecting ${{\rm{NH}}}_{3}$ gas (black circle), ${{\rm{NO}}}_{2}$ gas (blue circle) and ${{\rm{H}}}_{2}{\rm{S}}$ gas (red circle). And the sensor recovers to the initial state as the evacuation of mixed gas (green circle)

In Eq. (5), ${\omega }_{\pm }$ is the physical observable that has been used in our sensing study. In the regime of $\gamma \le \gamma ^{\prime}$, when $\gamma$ is in the proximity of $\gamma^{\prime}$, we define $\varepsilon =|\gamma -\gamma ^{\prime} |$ as the perturbation strength. Accordingly, the frequency splitting of these two peaks can be determined as $\Delta \omega =|{\omega }_{+}-{\omega }_{-}|\approx 2\sqrt{(\gamma ^{\prime} +{\gamma }_{0}+{\gamma }_{c})\varepsilon }\propto {\varepsilon }^{1/2}$. Such the square root dependence essentially amplifies the small perturbation, which is the basic mechanism for achieving ultra-sensitive sensing in this study. Such the square root behavior is originated from the underlying EP degeneracy of the eigenfrequencies. Figure 4e confirms that the frequency difference $\Delta \omega$ demonstrates a square root behavior as a function of $\varepsilon$.

As the system approaches to EPs, excess quantum noise will broaden the linewidth, which subsequently leads to an apparent loss of the sensor’s exceptional precision^43^. In our system, the transmission peak degeneracy does not occur at the EP, as shown in Fig. 2a, which avoiding excess quantum noise caused by non-orthogonality of the mode spectrum^29,37,38^. We further calculated the Petermann factor theoretically and measured it experimentally as a measure of non-orthogonality to quantify the linewidth broadening^44–46^. In the experimental domain, the Petermann factor is ranged from 1.1872 to 1.4519. Although the linewidth is broadened inevitably, the Petermann factor does not reach the level of divergence. More details about it can be found in the Supplementary materials S6.

### Sensing performance

In this section, we demonstrate sensing performance of the developed new SAW gas sensors. The red dots in Fig. 4a represent the average response times from split transmission peaks to single peak with different ${{\rm{H}}}_{2}{\rm{S}}$ gas concentrations. When the concentration of ${{\rm{H}}}_{2}{\rm{S}}$ gas was exceeded 2 ppm, the response of the sensor was extremely fast (e.g., within 10 s), showcasing the excellent response speed to tiny amounts of targeted gas molecules. As the ${{\rm{H}}}_{2}{\rm{S}}$ gas concentration decreased, the average response time gradually increased. Even at very low concentrations, such as 0.1 ppm, the sensor maintained a good response. However, it took much more time (e.g., over ten minutes) for the transmission peaks to merge. As well, we measured the frequency splitting with different ${{\rm{H}}}_{2}{\rm{S}}$ concentrations at a fixed response time (20 s), as shown by the yellow quadrilaterals in Fig. 4a. When the ${{\rm{H}}}_{2}{\rm{S}}$ concentration exceeded 2ppm, the response of our sensor was very fast. After 20 s, only a single peak in the transmission spectrum could be observed ($\Delta \omega =0$). As the ${{\rm{H}}}_{2}{\rm{S}}$ concentration decreased, the measured frequency splitting gradually increased. These results can be fitted as the calibration curve (the blue dashed line in Fig. 4a) which can be used to determine the ${{\rm{H}}}_{2}{\rm{S}}$ concentration of an unknown sample.

Figure 4b shows the robustness of our sensor at different environment temperatures. In the transmission peaks splitting regime, as the temperature was gradually increased, the global transmission spectrum exhibited a blue shift, while the frequency difference between the two peaks remained largely unchanged. Since our sensor is intrinsically self-referenced, the sensing relies on the measurement of frequency differences between these two peaks. Thus, the global frequency drift caused by temperature variation (or in other cases with different external sources) would not lead to misjudgment in gas detection. However, for the conventional SAW delay line gas sensors, as they are relying mainly on the measurement of the individual frequency shift, therefore, they do not possess such the advantage. For example, as the gas concentration or the temperature is increased, the transmission spectrum exhibits similar blue shifts^47,48^, thus it is difficult to judge whether the frequency shift is originated from the growth of detected gas or it is merely due to the increase of environment temperature.

${{\rm{SnO}}}_{2}$ film is a universal sensing material, which is well-responded to ${{\rm{H}}}_{2}{\rm{S}}$ gas molecules, and this property makes our sensor having excellent selectivity to detect ${{\rm{H}}}_{2}{\rm{S}}$ in a mixed gas, as demonstrated in Fig. 4c. We firstly continuously injected ${{\rm{NH}}}_{3}$ (which is belonged to reducing gas) into the reaction chamber. After about 300 s, although the concentration of ${{\rm{NH}}}_{3}$ gas reached about 1500 ppm, the transmission spectrum did not show apparent changes. Next, we subsequently injected an oxidizing gas of ${{\rm{NO}}}_{2}$ to reach the similar concentration, the transmission spectrum still did not show apparent changes. Ultimately when we injected minor amount of ${{\rm{H}}}_{2}{\rm{S}}$ gas (about 0.5 ppm), the sensor showed rapid responses and these two separated peaks were quickly coalesced into one within about 90 s. After all the mixed gas was released, the sensor was gradually recovered to its initial state, demonstrating the excellent recovery ability of our sensor. More selectivity test can be found in Fig. S7 in Supplementary materials.

## Conclusion

Additional loss in the resonator, which causes linewidth broadening and reduces the resolvability of conventional sensor devices, is typically regarded as a drawback for precision sensing and is often aimed to minimized as much as possible^28,49^. However, in this work we actively utilized the additional loss which was brought by a sensitive thin film to achieve an ultrasensitive sensing performance. Taking the gas sensor as an example, the introduction of the additional loss in two coupled SAW resonators structure formed a passive PT-symmetric system. When a tiny amount of ${{\rm{H}}}_{2}{\rm{S}}$ molecules were injected, the device exhibited a process in which two peaks were merged into one single peak in the transmission spectrum. When the system was in the PT exact phase regime, and approached the coalescence point of two transmission peaks, their frequency difference was proportional to the square root of perturbation strength, resulting in significantly enhanced sensitivity compared to the conventional linear response of the delay-line SAW sensor. In addition, our proposed SAW sensor exhibited an outstanding robustness against temperature variations and exhibits a good selectivity to ${{\rm{H}}}_{2}{\rm{S}}$. This novel design concept for achieving good sensing performance can be easily extended to other types of non-Hermitian sensors and implement more functions, such as mechanical/biological/chemical sensing, wireless sensor, the more sensitive sensor based on higher-order EP, etc. In addition, such newly proposed SAW system provides an excellent platform to realize non-Hermitian system at micro/nano scales. The developed SAW system also allows for convenient tuning of the resonant frequency and coupling strength. This enables the fabrication of various non-Hermitian MEMS devices to explore intriguing non-Hermitian physics and develop high-performance devices.

## Methods

### Experimental set-ups and measurement

The SAW chip was composed of IDTs, Bragg mirrors and sensitive thin film fabricated on a 128° Y-cut ${{\rm{LiNbO}}}_{3}$ substrate. Here the ${{\rm{LiNbO}}}_{3}$ substrate is selected because of its higher electromechanical coupling coefficient and lower propagation loss. Furthermore, the additional loss introduced by the sensitive layer is proportional to the electromechanical coupling coefficient of substrate (see Eq. (7)), which means that the high electromechanical coupling coefficient of the ${{\rm{LiNbO}}}_{3}$ substrate could induce a stronger response of the sensor. Both the IDTs and Bragg mirrors were composed of 100 nm thick and 4.9 μm wide aluminum electrodes, and the pitch of both was 9.8 μm. The numbers of electrodes of input and output IDTs were set as 60. The Bragg mirrors were shorted-circuited gratings. To compensate the frequency deviation caused by the deposition of the ${{\rm{SnO}}}_{2}$ thin film, the optimized numbers of electrodes for the Bragg mirrors near the input port, in the middle and near the output port were 40, 60 and 50, respectively. The widths of two SAW resonators were $500\upmu {\rm{m}}$ and $402\upmu {\rm{m}}$.

A $200{\rm{nm}}$ thick, $300\upmu {\rm{m}}$ width ${{\rm{SnO}}}_{2}$ thin film was deposited onto the surface of the resonator near the input port using the magnetron sputtering technique. In the fabricating process, the base pressure before deposition was below $9\times {10}^{-4}{\rm{Pa}}$ and during depositing it was kept at $2{\rm{Pa}}$. The sputtering power was set at 100 W. The device with deposited thin film was annealed at a temperature of $773.15{\rm{K}}$ for 2 h.

Our experiment setup was composed of a gas reaction chamber (with wall thickness 1 cm, size $1{\rm{dm}}\times 1{\rm{dm}}\times 1{\rm{dm}}$). The lid on the top of the reaction chamber was openable. On the side wall, there were two valves used for injecting and evacuating gas, and some electrodes for connecting sample and measurement instrument. The prepared SAW chip (being wire-bonded to the printed circuit board) was connected to a network analyzer for detecting SAW signals. The photo of experimental setup can be found in Supplementary materials Fig. S8. In experiments, we sealed the reaction chamber and injected a predetermined amount of ${{\rm{H}}}_{2}{\rm{S}}$ gas. A LabVIEW program was developed to control the network analyzer and measure the ${{\rm{S}}}_{21}$ (transmissivity equals to ${|{{\rm{S}}}_{21}|}^{2}$) of the SAW device at the time cycle of one second. When the transmission spectrum was no longer changed within at least 10 min, we then opened the air valve and used an air pump to inject air and removed the detected gas in the chamber. After the detected gas was released, the device was returned to its initial state and could be used to carry out the next experiment. The sensing performance tests of the conventional delay line sensor used the same method.

### Simulation

Commercial software COMSOL Multiphysics was applied to determine the structural parameters of the device and simulate the sensing experiments, based on a 2D cross-section module of SAW device. We applied solid mechanics module and electrostatics modules to carry on the simulation, and considered the coupling of strain field and electrostatic field due to the piezoelectric effects. The whole device except the top surface was surrounded by the perfectly matched layers (PML) to absorb the radiated waves. The Bragg mirrors were set as the floating potential. Mechanical damping module and dielectric loss module were introduced in area where the thin film was deposited to simulate the additional loss from the thin film.

After the number of electrodes in the Bragg mirror have been determined, the parameters in Eq. (4) are dependent on the specific characteristics of sensitive layer and how it introduces loss. In simulation, $\varGamma$ was used as the loss factor in mechanical damping module and dielectric loss module of piezoelectric substrate in the area where the thin film was deposited. We applied analytical expression Eq. (4) to calculate the transmission spectrum and fitted it to the simulated transmission spectrum at different loss factor. By adjusting the parameters in Eq. (4), we aimed to make the evolution of transmission with respect to $\gamma$ as close as possible to the simulation (the analytical results and simulated results have minimum mean variance). Finally, the relevant parameters could be determined as ${\gamma }_{0}=8\times {10}^{3}{\rm{Hz}}$,${\gamma }_{c}=1.2\times {10}^{4}{\rm{Hz}}$,$\kappa =2.93\times {10}^{5}{\rm{Hz}}$. Therefore, using ${\kappa }^{2}-\frac{{\gamma^ {\prime} }^{2}}{2}-\gamma^ {\prime} ({\gamma }_{0}+{\gamma }_{c})-{({\gamma }_{0}+{\gamma }_{c})}^{2}=0$, the additional loss at critical state was evaluated to be $\gamma^ {\prime} =0{\rm{.393MHz}}$.

Actually, our sensitive sensing effect can be achieved as other available piezoelectric substrates are chosen or at other operating frequencies, as long as the coupled-resonators structure is elaborately designed. To demonstrate the universality of our architecture, we represent the simulation results of the EP effects on the quartz substrate and at higher SAW frequencies in Supplementary materials, as is shown in Figs. S9, S10.

## Supplementary information

Supplementary materials for Harnessing Exceptional Points for Ultrahigh Sensitive Acoustic Wave Sensing
