# Supplementary material for: Harnessing exceptional points for ultrahigh sensitive acoustic wave sensing
Source: Microsyst Nanoeng. 2025 Mar 7;11:44. doi: 10.1038/s41378-024-00864-5 (PMC11889215; doi:10.1038/s41378-024-00864-5)
Supplement: Supplementary file 1 — Supplementary materials for Harnessing Exceptional Points for Ultrahigh Sensitive Acoustic Wave Sensing [file 41378_2024_864_MOESM1_ESM.docx]

*Supplementary materials* for

**Harnessing Exceptional Points for Ultrahigh Sensitive Acoustic Wave Sensing**

Xingyu Lu, Yang Yuan, Fa Chen, Xiaoxiao Hou, Yanlong Guo, Leonhard Reindl, Yongqing Fu, Wei Luo and Degang Zhao

**S1**  **leads to the annihilation of EP**


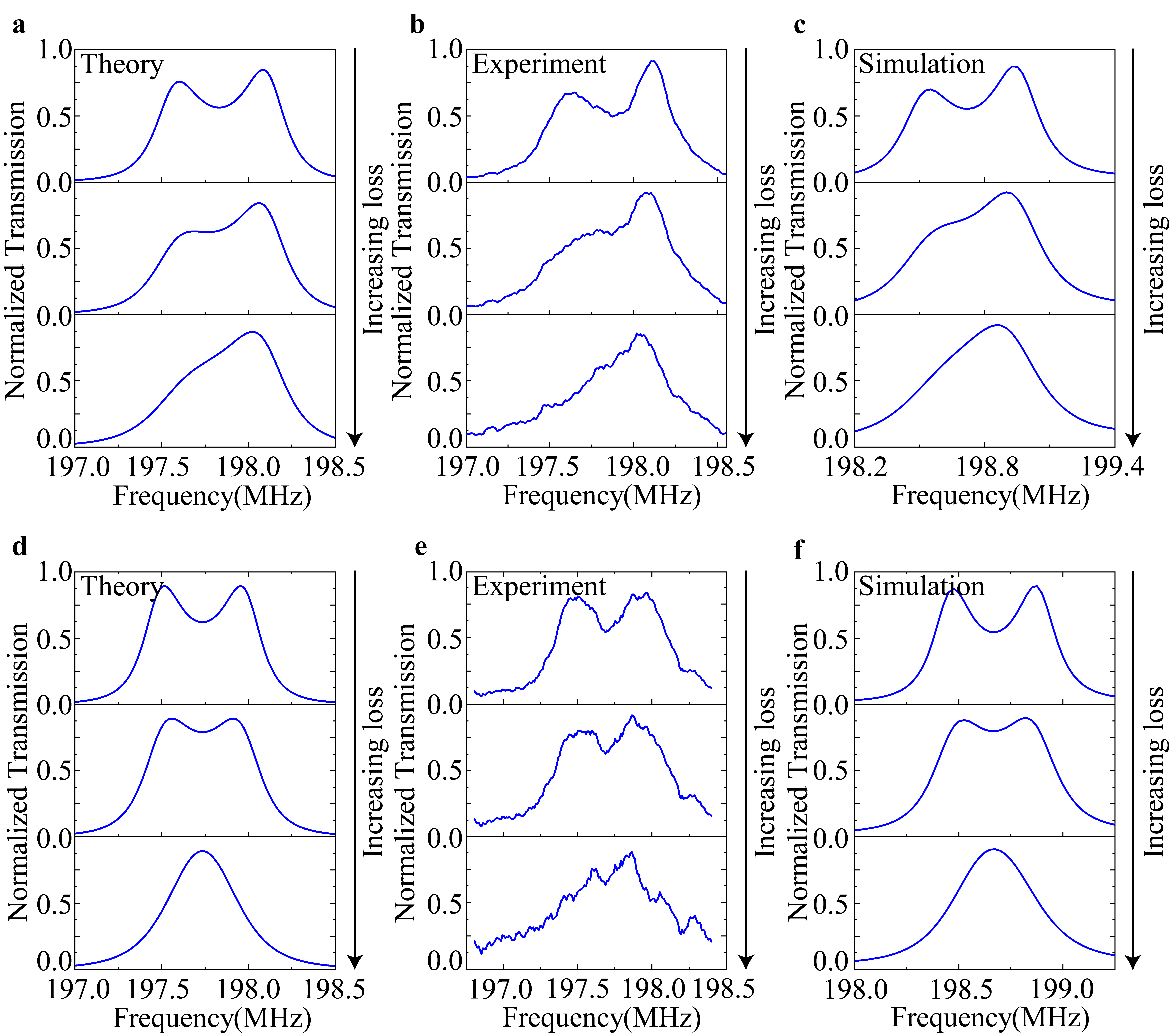


**Fig. S1.** The evolution of the two transmission peaks obtained by coupled mode theory, experiment and simulation, as the increase of additional loss, with $\omega_{1}=0.999\omega_{2}$ (a-c) and $\omega_{1}=\omega_{2}$ (d-f).

The detuning of resonator frequencies due to the introduction of the thin film is detrimental to the sensing performance. Only $0.1\text{\%}$ frequency shift is introduced in $\omega_{1}$ results in apparently uneven height transmission peaks, as is shown in Fig. S1(a-c). With the increase of additional loss $\gamma$, one peak experience more significant dissipation and rapidly vanishes, while the other peak suffers less loss and it almost keeps unchanged. Furthermore, the final presented single peak may mislead observer that it forms by the merger of two peaks and the system is in the PT-broken phase regime. Actually, it is not. To compensate the frequency shift induced by the thin films, we carefully adjust the structure of the resonator without deposited thin film. In the simulation, we gradually add the number of electrodes of the Bragg mirror near the output IDT to achieve $\omega_{1}=\omega_{2}$. Ultimately, the optimal number of electrodes of the left, middle and right Bragg mirrors are 40, 60, and 50, respectively. After optimization, two distinct peaks having nearly even height in transmission spectrum can be observed, as is shown in Fig. S1(d-f). And as the increase of additional loss, these two peaks gradually get closer to each other, and finally merge into one single peak.

**S2 Estimate additional loss**


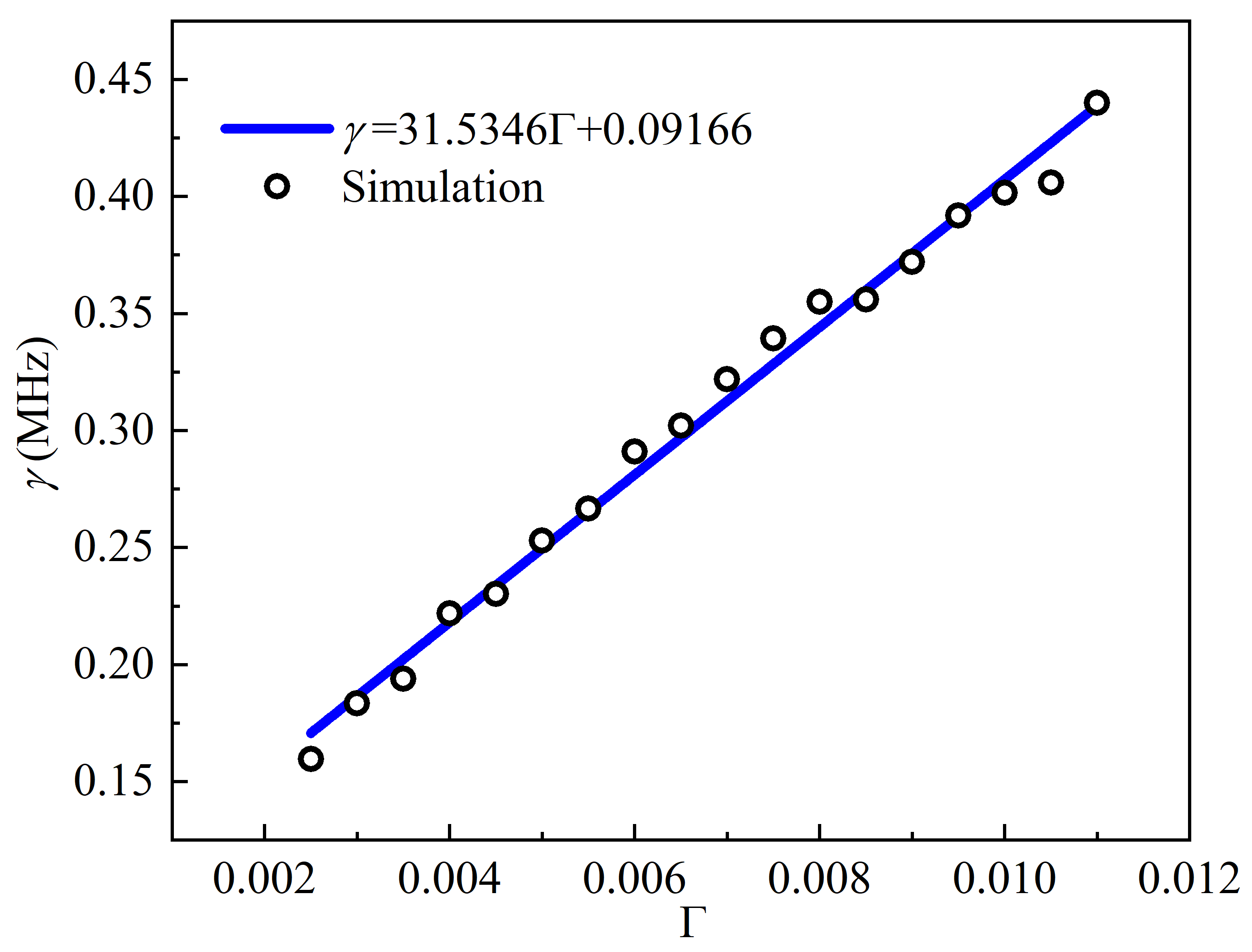


**Fig. S2.** The relationship between SAW attenuation $\Gamma$ and the additional loss $\gamma$ in Hamiltonian.

One key point in the device design lies in accurately evaluating the additional loss brought by the thin film. Firstly, according to the Eq. 7 in main text, it is obvious when $\sigma_{0}=\sigma_{0r}$, the SAW attenuation reaches its maximum value. In the pre-experiment, we deposit a single $SnO_{2}$ thin film (with the same size of the thin film in sensing experiment, but without Bragg mirrors) onto the $\text{LiNbO}_{3}$ substrate. After injecting $\text{H}_{2}\text{S}$ gas, with the evolution of time, the sheet conductivity of the thin film changes. We measure the sheet conductivity and corresponding transmission spectrum. When the transmission peak reaches its minimum, i.e. the attenuation reaches maximum, the corresponding sheet conductivity is the parameter $\sigma_{0}$. In simulation, $\Gamma$ is used as the loss factor in mechanical damping module and dielectric loss module of piezoelectric substrate in the area which is deposited thin film to calculate the transmission spectrum. Next using analytical expression Eq. 4 in main text and comparing it to the simulated results, we can fit the function between $\Gamma$ and $\gamma$: $\gamma=31.5346\text{ }\Gamma+0.09166 \text{(MHz)}$, as is shown in Fig. S2, which reveals the approximate linear relationship between $\Gamma$ and $\gamma$. The small constant term may stem from energy dissipation in the thin film individually. Both the proportional coefficient and constant term are relevant to the size of film, the raw materials of film and the piezoelectric substrate. Notably, a wider thin film (along the direction of acoustic wave propagation) may introduce more frequency detuning and excessive loss, which may broaden the linewidth and diminish the resolvability of the transmission peaks. Conversely, too narrow thin film may not generate sufficient loss. Through pre-experiment and simulation, we reasonably evaluate additional loss and frequency detuning, and optimize the width of thin film to be $\text{300 μm}$.

**S3 Pre-experiment to determine the appropriate sputtering time.**


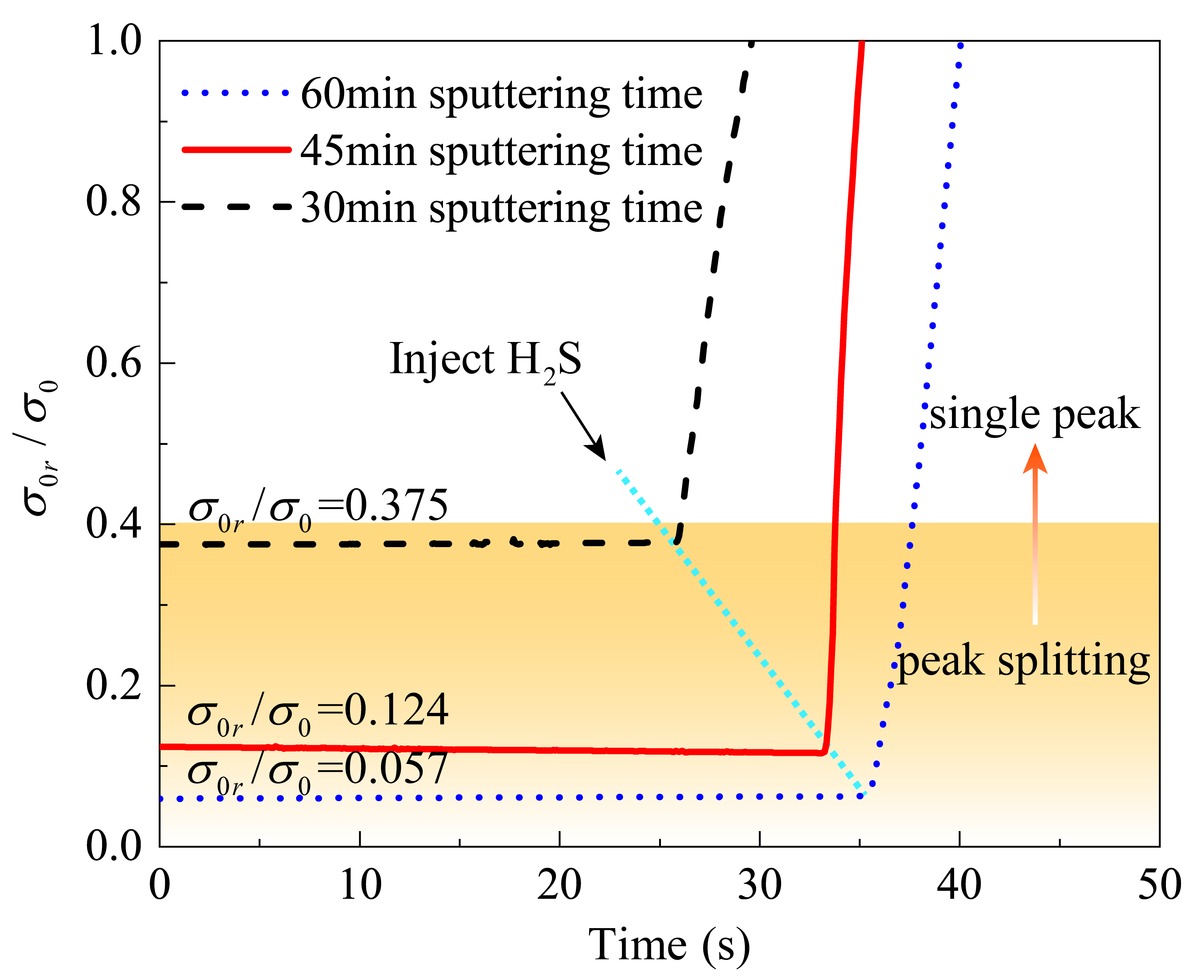


**Fig. S3.** The relationship between the initial state of thin films and sputtering time. For different magnetron sputtering time, $\text{SnO}_{2}$ thin films have different stable initial $\sigma_{0r}/\sigma_{0}$. After injecting $H_{2}S$ gas, $\sigma_{0r}/\sigma_{0}$ of all the thin films increases rapidly.

The $SnO_{2}$ thin film was deposited using the magnetron sputtering technique, producing an initial sheet conductivity and initial additional loss, which will determine the initial state of system (in PT exact phase or PT-broken phase). For effectively achieve sensing, the initial additional loss must be smaller than $\gamma'$ in Eq. 5 in main text. We observed that when maintaining constant pressure and temperature, the initial sheet conductivity of the thin film is primarily influenced by the sputtering time. Fig. S3 shows the measured sheet conductivity of the $SnO_{2}$ thin film with 30, 45 and 60 minutes of sputtering time. It clearly embodies that the longer the sputtering time, the lower the initial sheet conductivity of $\sigma_{0r}$. After injecting 1 ppm $\text{H}_{2}\text{S}$ gas, the sheet conductivities of the thin film were increased rapidly, due to the high sensitiveness of $SnO_{2}$ thin film to $\text{H}_{2}\text{S}$ gas.

**S4 The delay line gas sensor**


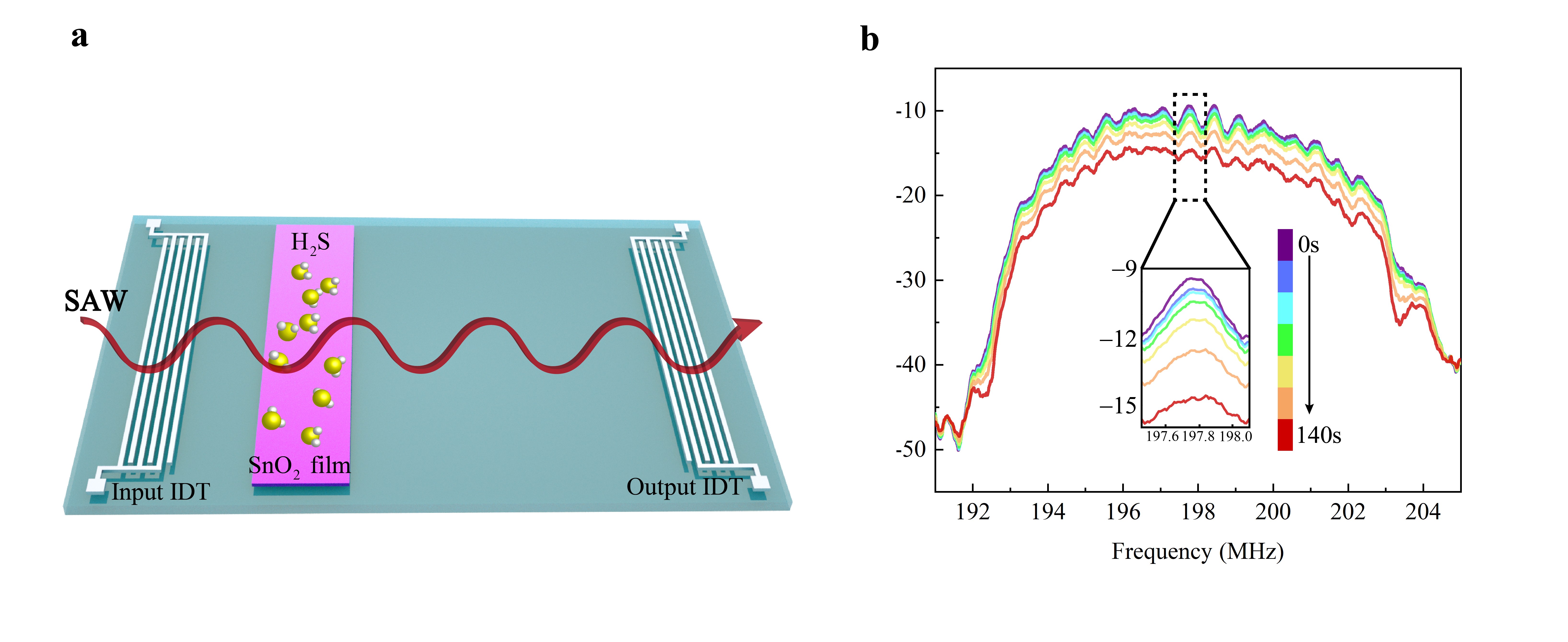


**Fig. S4.** The delay line gas sensor. (a) Schematic diagram of a delay line gas sensor. (b) The evolution of the transmission spectra over time for the delay line gas sensor as the injection of $0.4\text{ppm}$ $\text{H}_{2}\text{S}$ gas.

To compare sensing performance, we remove all Bragg mirrors, forming a delay line gas sensor, as is shown in Fig. S4a. After injecting $0.4\text{ppm}$ $\text{H}_{2}\text{S}$ (as the same gas concentration injected in EP-based gas sensor in the main text), the passband ripple exhibits almost indistinguishable frequency shifts, as is shown in Fig. S4b. Such a blurred phenomenon can hardly be used to accurately detect $\text{H}_{2}\text{S}$ gas. The injection of gas brings about tiny variation in the conductivity of the thin film, subsequently cause small change in wave number $\Delta k$. Actually, the conventional gas sensor utilizes $\mathrm{Re}(\Delta k/k)$, while our EP-based gas sensor utilizes $\mathrm{Im}(\Delta k/k)$. That small $\Delta k$ causes little frequency shift. To achieve a more substantial frequency shift, conventional gas sensors typically employ wider thin films or introduce larger amounts of target gases, often in the range of hundreds of ppm. However, for our EP-based gas sensor, although $\Delta k$ is small, as long as the corresponding additional loss $\gamma$ approaches critical value $\gamma'$, the transmission spectrum will exhibit significant change due to the underlying EP nature.

**S5 The shift of resonant frequency**

Mass loading, elastic loading, and electric effect will all affect the wave velocity of SAWs, resulting in a slight shift in the resonant frequency $\omega_{0}$ of two resonators^1^: $\frac{\delta\omega}{\omega_{0}}\cong\frac{\delta v}{v_{0}}=-C_{m}\omega_{0}\delta(\rho_{s})+4C_{e}\omega_{0}\delta(hG')-\frac{K^{2}}{2}\delta(\frac{1}{1+(\frac{v_{0}c_{s}}{\sigma_{s}})^{2}})$, where $-C_{m}\omega_{0}\delta(\rho_{s})$ represents mass loading, $4C_{e}\omega_{0}\delta(hG')$ represents elastic loading, and $-\frac{K^{2}}{2}\delta(\frac{1}{1+(\frac{v_{0}c_{s}}{\sigma_{s}})^{2}})$ represents electric effect. When all these effects are considered, a frequency shift term $\delta\omega(\gamma)$ can be introduced in the Hamiltonian of the coupled resonators (Eq. 1 in main text):

$H_{0}=\left(\begin{matrix} \omega_{0}\text{+}\delta\omega(\gamma)-i\gamma_{0}-i\gamma& \kappa\\ \kappa& \omega_{0}\text{+}\delta\omega(\gamma)-i\gamma_{0} \end{matrix}\right)$. (S1)

Considering the value of $\delta\omega(\gamma)$ to repeat transmission analysis process in the main text, the evolution of frequency is plotted in Fig. S5 (the red line), which agrees well with the experimental results shown in Fig. 3d. Obviously, the EP effect (transmission peak degeneracy) is not dramatically changed by this offset.


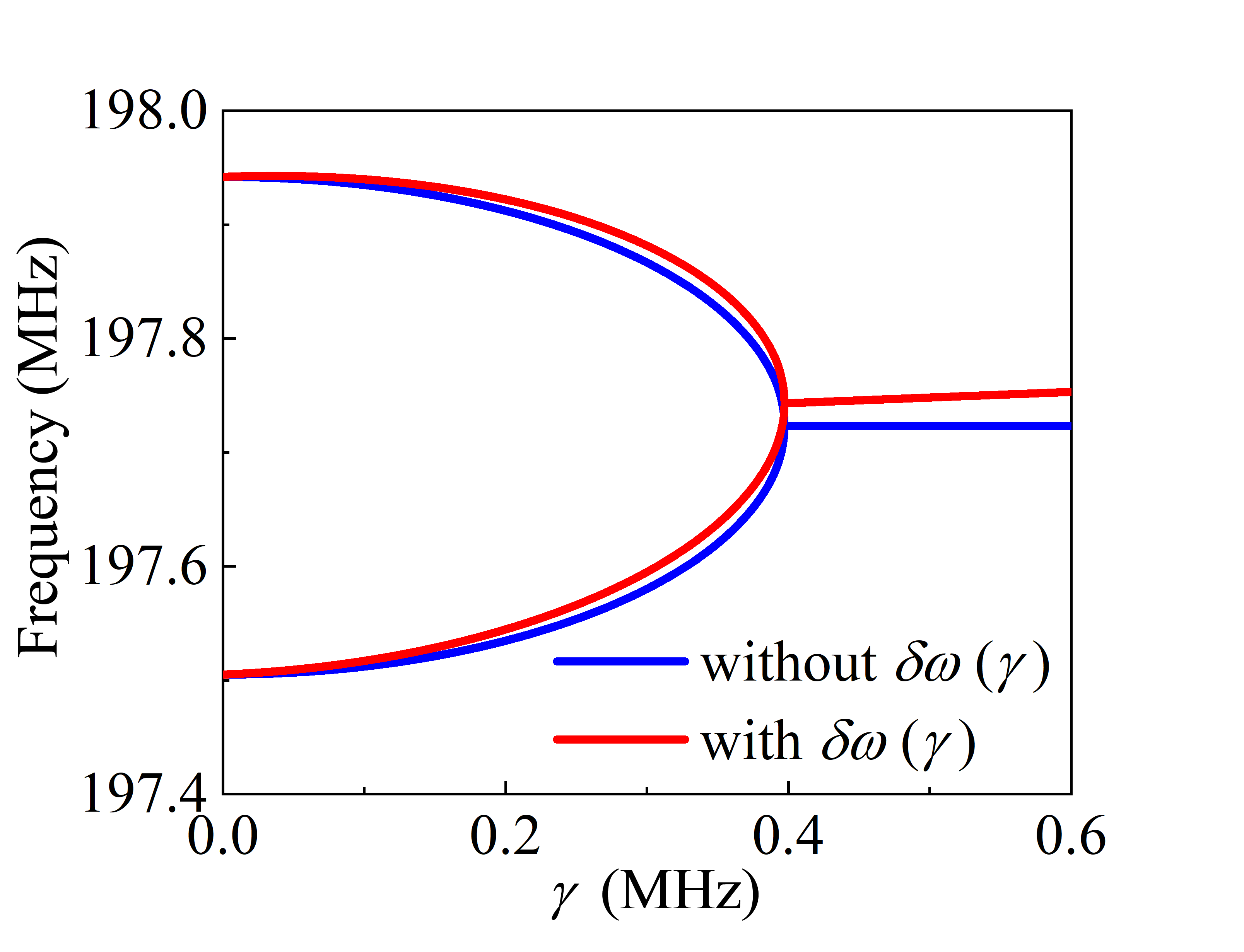


**Fig. S5.** The comparisons of the evolution of transmission peaks’ frequency with (red curve) and without (blue curve) $\delta\omega(\gamma)$.

**S6 Petermann factor**

The Petermann factor, as a measure of the non-orthogonality, has been introduced to quantify the linewidth broadening resulted from excess quantum noise. Theoretically, in the two-dimensional Hilbert spaces, the Petermann factors can be defined as^2^:

$K=\frac{1}{2}(1+\frac{\text{Tr}(H^{\dagger}H)}{\left|\text{Tr}(H^{2})\right|})=\frac{1}{2}(1+\frac{4\kappa^{2}+\gamma^{2}}{\left|4\kappa^{2}-\gamma^{2}\right|})$, (S2)

where $H=H_{0}-Tr(H_{0})/2$. In the experiments, the Petermann factor was calculated by the ratio of the measured linewidth to the theoretical linewidth (which can be obtained from Eq. (4) in main text)^3^. The comparison results are presented in Fig. S6. In the experimental domain, the Petermann factor was ranged from 1.1872 to 1.4519, which was very far from divergence.


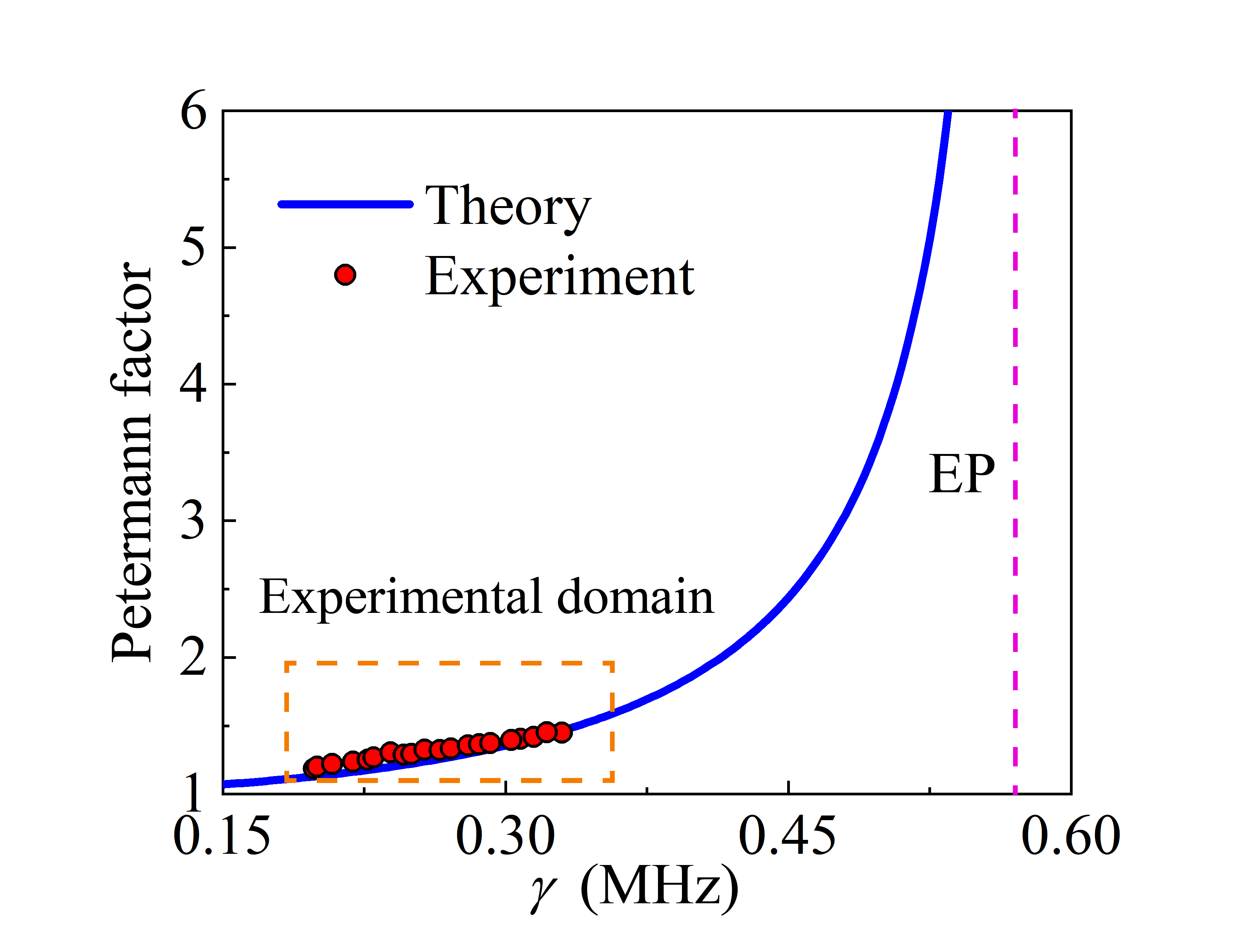


**Fig. S6.** The Petermann factors calculated theoretically and measured experimentally. In the experimental domain, the Petermann factor is ranged from 1.1872 to 1.4519.





**Fig. S7.** Response of sensor to some other test gases.


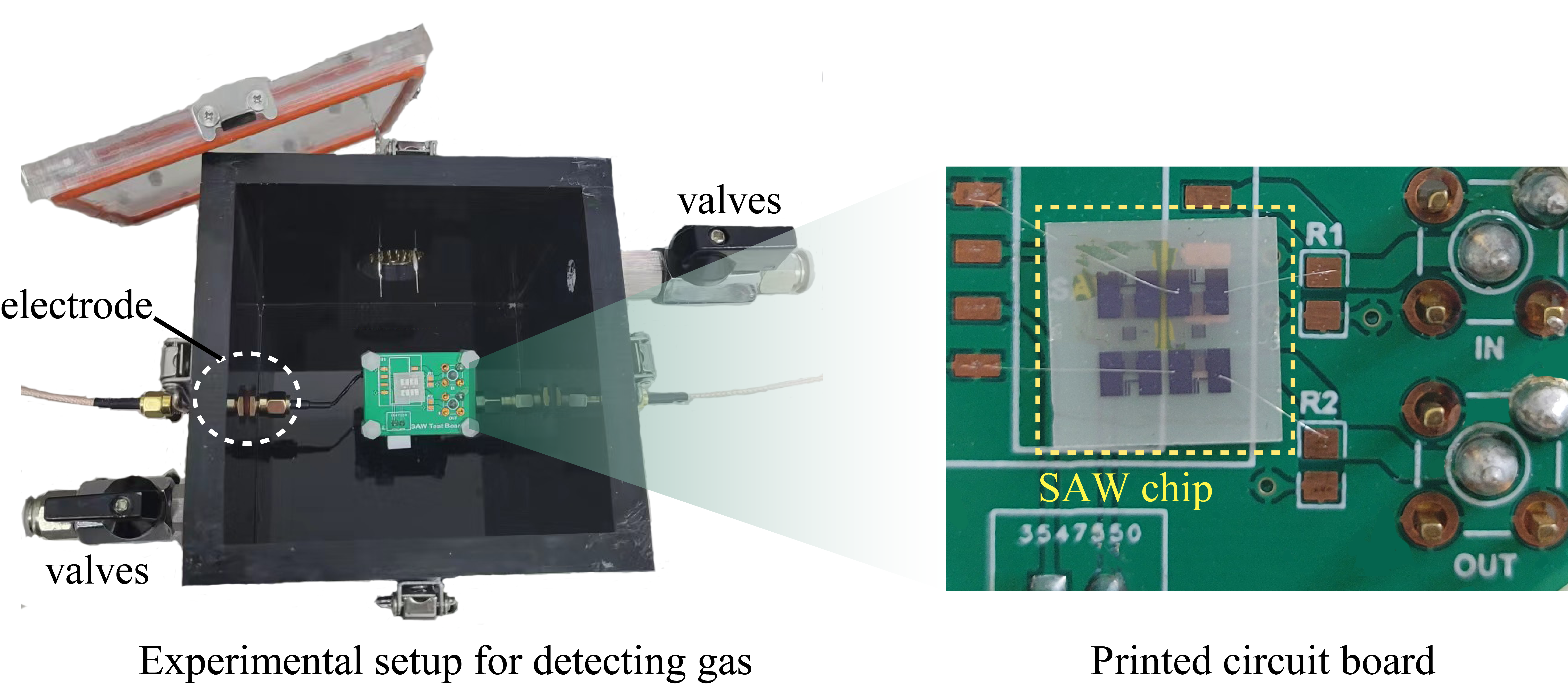


**Fig. S8.** The photo of the experimental setup for gas sensing.


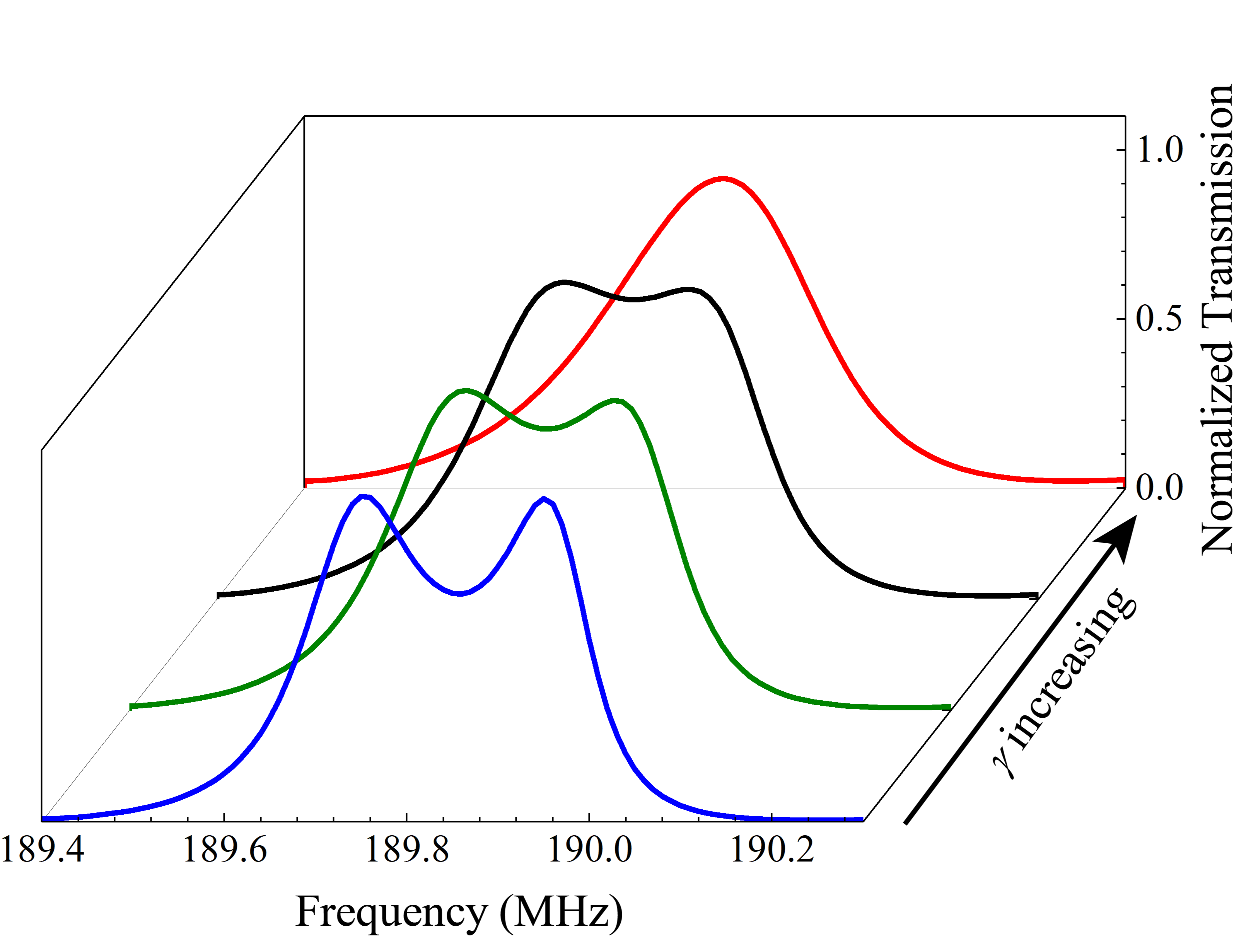


**Fig. S9.** EP effect realized on a quartz substrate. The number of electrodes for coupling two SAW resonators is increased to 140, with all the other structural parameters kept unchanged as those of the $\text{LiNbO}_{3}$ substrate.


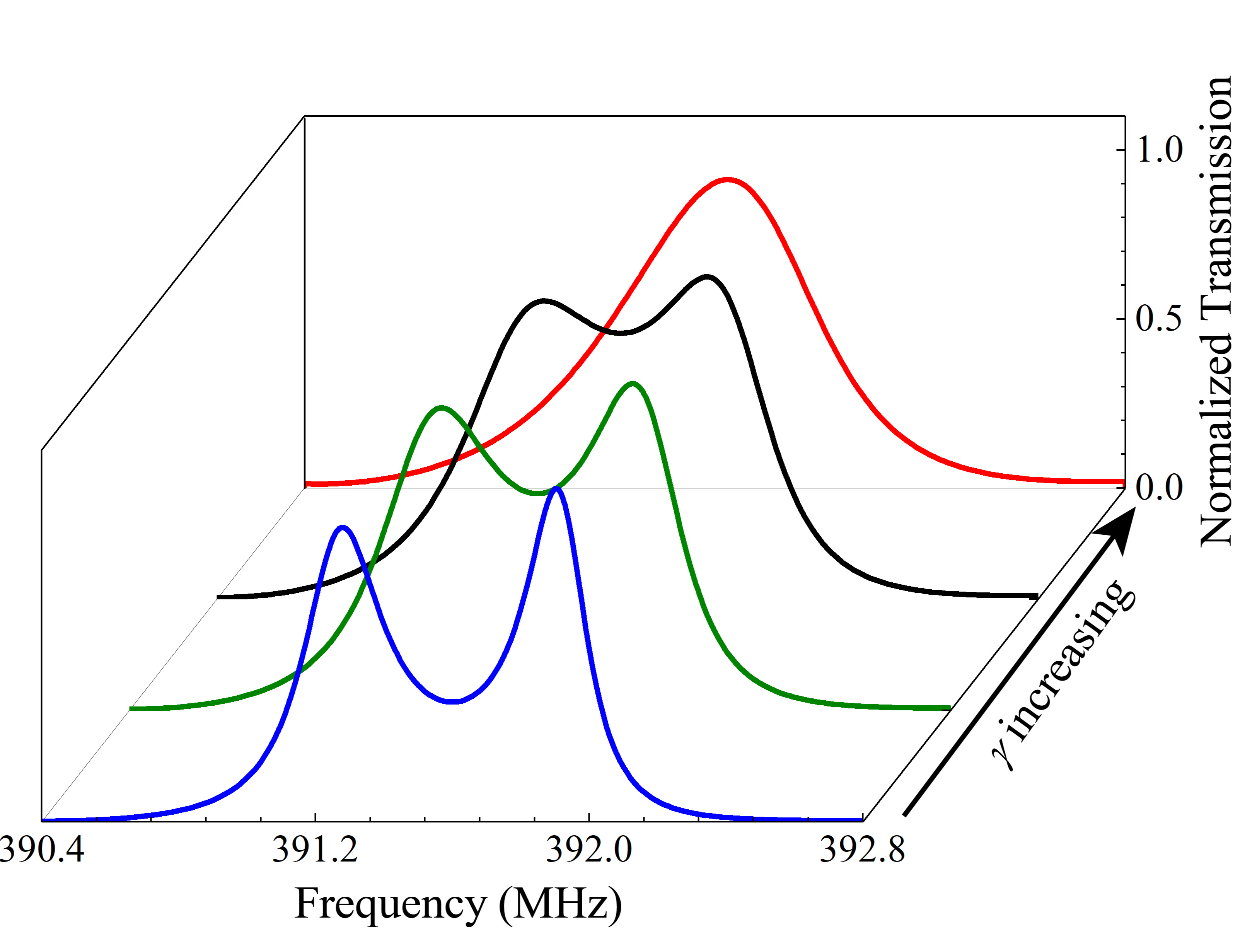


**Fig. S10.** EP effect achieved at the higher SAW frequencies. The width of IDT and reflecting gratings have been reduced to half of the value for the devices reported in the main text. The other structural parameters are kept unchanged but the number of electrodes for coupling two SAW resonators are increased to 120.

**References**

1. Galipeau, J. D. et al. Theory, design and operation of a surface acoustic wave hydrogen sulfide microsensor. *Sens. Actuators B Chem.* **24**, 49-53 (1995).

2. Wang, H., Lai, YH., Yuan, Z. et al. Petermann-factor sensitivity limit near an exceptional point in a Brillouin ring laser gyroscope. *Nat Commun* **11**, 1610 (2020).

3. Schomerus, H. Excess quantum noise due to mode nonorthogonality in dielectric microresonators. *Phy. Rev. A.* **6**, 061801 (2009).
